# Anti-Aggregating Effect of the Naturally Occurring Dipeptide Carnosine on Aβ1-42 Fibril Formation

**DOI:** 10.1371/journal.pone.0068159

**Published:** 2013-07-03

**Authors:** Alessandra Aloisi, Amilcare Barca, Alessandro Romano, Sara Guerrieri, Carlo Storelli, Rosaria Rinaldi, Tiziano Verri

**Affiliations:** 1 National Nanotechnology Laboratory (NNL) of Consiglio Nazionale delle Ricerche (CNR) – Istituto Nanoscienze Lecce, Lecce, Italy; 2 Department of Biological and Environmental Sciences and Technologies (DiSTeBA), University of Salento, Lecce, Italy; 3 Mathematics and Physics “*E. De Giorgi*” Department, University of Salento, Lecce, Italy; Universitat Autònoma de Barcelona, Spain

## Abstract

Carnosine is an endogenous dipeptide abundant in the central nervous system, where by acting as intracellular pH buffering molecule, Zn/Cu ion chelator, antioxidant and anti-crosslinking agent, it exerts a well-recognized multi-protective homeostatic function for neuronal and non-neuronal cells. Carnosine seems to counteract proteotoxicity and protein accumulation in neurodegenerative conditions, such as Alzheimer’s Disease (AD). However, its direct impact on the dynamics of AD-related fibril formation remains uninvestigated. We considered the effects of carnosine on the formation of fibrils/aggregates of the amyloidogenic peptide fragment Aβ1-42, a major hallmark of AD injury. Atomic force microscopy and thioflavin T assays showed inhibition of Aβ1-42 fibrillogenesis *in vitro* and differences in the aggregation state of Aβ1-42 small pre-fibrillar structures (monomers and small oligomers) in the presence of carnosine. *in silico* molecular docking supported the experimental data, calculating possible conformational carnosine/Aβ1-42 interactions. Overall, our results suggest an effective role of carnosine against Aβ1-42 aggregation.

## Introduction

Carnosine (β-Ala-L-His) is a bioactive dipeptide endogenously abundant in the central nervous system (CNS) [1]. High rates of carnosine synthesis are thought to occur in glial cells (oligodendrocytes and astrocytes), but not in neurons, that are conversely thought to mainly receive carnosine from glial cells [2]–[6]. Carnosine is known to operate as intracellular pH buffer modulator, Zn/Cu ion chelator, and antioxidant, aldehyde-scavenger, antiglycating and anti-crosslinking agent for proteins [1], [7]–[15]. In the CNS, it is assumed to work as a multi-functionally homeostatic and protective molecule for neuronal and non-neuronal cells, bringing inherent benefits in terms of counteracting to neurodegenerative conditions [14]–[20]. Carnosine has been analyzed as a specific metabolic tool against neuronal toxic effects, such as those arising from age-related proteotoxicity or from pathophysiological pathways leading to altered protein accumulation [1], [18], [21]–[29], and its protective effects against aberrant amyloid peptides have been tested in various mammalian tissues and cells [28], [30], [31]. Interestingly, carnosine has also been investigated in tissues and fluids from patients with well-known neurodegenerative conditions/pathologies, such as Parkinson’s Disease, Freiderich’s ataxia and Alzheimer’s Disease (AD) [1], [11], [26], [32]–[37]. In the context of AD, the genes involved in carnosine metabolism have also been investigated. In particular, the activity of the brain-specific carnosinase has been shown to be altered in fluids from patients with AD dementia [38] and, more recently, this enzyme has been validated as a novel biomarker in the cerebro-spinal fluid for staging early AD [39]. Furthermore, the mRNA of PEPT2 [40], a carrier protein involved in transmembrane transport of carnosine, has been studied as a marker for differential staging of AD progression in mammalian models [41].

A key feature in AD pathogenesis is the excess formation/accumulation of amyloid fibrils and plaques. The predominant portion of the AD neuritic amyloid formations consists of the peptide fragment Aβ1-42, produced physiologically by the amyloid precursor protein, which readily associates into soluble oligomers, required for AD-related neurotoxicity onset [42], [43]. The aberrant accumulation of Aβ1-42 is directly involved in the escalation of the neuronal injuries typical of AD [44]–[46], and its plasma levels strictly correlate to the severity of the disease [43], [47]. The self-associating Aβ1-42 peptides form nucleation centers [48] from where the amyloid fibrils can quickly grow, contributing primarily to form the AD-related senile plaques [49]–[54]. The huge tendency of Aβ1-42 to display fibril formation has clearly been demonstrated by *in vitro* assays [55]. Also, the structure-neurotoxicity relationships of Aβ1-42 fragments have been investigated in depth with respect to morphology and polymerization state of aggregates and fibrils [43], [55], [56].

Currently, the inhibitory activity of small molecules (small peptides included) able to break down the structural organization of soluble or aggregating Aβ1-42 in the fibrillogenesis process is under investigation [57], [58] with the aim of identifying novel inhibitors of Aβ1-42 aggregation and toxicity, a major topic in AD research [59]. In this context, we considered the direct effects of carnosine, a naturally occurring dipeptide in nervous cells, on the fibrillogenesis process of the Aβ1-42 fragment.

## Materials and Methods

### Materials

Aβ1-42 amyloidogenic peptide fragment corresponding to the human amino acid sequence, carnosine (β-Ala-L-His), β-alanine, L-histidine and Thioflavin T (ThT) were purchased (reagent grade) from Sigma Aldrich (St. Louis, USA).

### Sample Preparation for Fibrillogenesis Assays

Aβ1-42 stock solution (100 µM) was prepared by dissolving the peptide fragment powder (two different lots from Sigma-Aldrich were used, namely lot n. 079K8729 and SLBC5079V) in sterile Milli-Q water, as previously reported [43], [58], [60]–[65]. Aliquots (5 µl) were lyophilized and stored at −20°C until use. For fibrillogenesis assays, Aβ1-42 lyophilized aliquots were routinely reconstituted in 50 mM Tris-HCl, pH 7.4 (5 µl) [61] to the original concentration of 100 µM. Solubilized Aβ1-42 was incubated in a water bath for 30 min at 37°C under gentle mixing, either alone or in the presence of carnosine (0.1, 1 and 10 mM) or hydrolysed carnosine (β-alanine and L-histidine, 10 mM each).

### Sample Adsorption for Fibrillogenesis Assays

Sample aliquots were removed from the water bath, diluted 1∶2 (5 to 10 µl) with 50 mM Tris-HCl, pH 7.4, and rapidly casted on freshly cleaved mica. After dehydration for 15 min at room temperature (RT: 23–26°C, relative humidity ∼40%) in a not hermetically covered box, samples were rinsed thrice with 50 µl Milli-Q water in order to remove salt and loosely bound molecules. Samples were taken to dryness in a gentle stream of nitrogen. Then, they were promptly imaged.

### Atomic Force Microscopy (AFM)

All images were recorded in air at RT using a Nanoscope VI Multimode Scanning Probe workstation (Digital Instruments, Santa Barbara, CA) operating in tapping mode with phosphorus doped silicon cantilevers, tip radius of 8 nm and a resonance frequency of 69–92 kHz (probe model R FESPA, Digital Instruments). Different scanner types were used (*Picoforce* and *E* –type, with xy range of 40 µm and 15 µm, respectively). Recording parameters varied with individual samples, hence consecutive shots were monitored before collecting images at sizes of 5, 2.5, 2.0 or 0.5 µm^2^, with the maximum 512×512 pixel resolution, and scan rate from 1 to 1.5 Hz.

### ThT Fluorescence Assays

ThT stock solution was prepared at a final concentration of 1.5 mM in Tris-HCl, pH 7.4. The solution was filtered through a 0.22 µm pore size filter and stored in the dark at 4°C for no longer than a week. To quantify Aβ1-42 aggregation state, a typical ThT fluorescence assay was conducted [66], [67]. Spectrofluorimetric measurements were performed by adding ThT to a 100 µM Aβ1-42 solution, in the presence (0.1, 1 and 10 mM) or absence of carnosine and hydrolysed carnosine (β-alanine and L-histidine, 10 mM each), under the fibril aggregation conditions described above. Briefly, fluorescence emission spectra of ThT incorporated into β-sheet amyloid structures are red-shifted [66]. Thus, binding of ThT micelles to growing fibrils results in enhanced ﬂuorescence signal [67]. ThT was incubated for 10 min with Aβ1-42 at the molar ratio of 1∶2, in the presence or absence of carnosine. Fluorescence was measured by a Varian Cary Eclipse spectrofluorometer (JVA Analytical Ltd, Dublin, Ireland), using excitation and emission wavelengths of 440 and 482 nm, respectively (slit widths 5 nm). Emission spectra were collected (between 450 and 560 nm). The emission spectrum of carnosine alone was subtracted, and emission data of peptide dispersions were normalized.

### Fibril Morphology Analysis

Structures heights were measured by using the *NanoScope* software v7.30 (Bruker, Mannheim, Germany). The roughness routine statistics was used in order to get peaks height data, expressed as the average distance between the five highest profile points and the mean data plane (R_pm_) over areas of 5 µm^2^. Images, acquired from at least 3 independent tests, were analysed for each experimental condition.

Measurements of contour lengths were performed manually on binary transformed images, using the ImageJ software v1.43 (National Institute of Health, Bethesda, MD; http://rsbweb.nih.gov/ij/), by truthfully tracing the backbone of the selected fragments. A total of at least 200 aggregates were measured from 6 separate AFM images for each experimental condition. Only fibril-like aggregates that could clearly be sized over 30 nanometers were included in the count [43], [55]. Numerical data were exported as ordinary ASCII files, tabulated and plotted using OriginPro 8 (OriginLab Corporation, Northampton, MA). All dimensional data were obtained from not processed images with respect of flatten or plane fit inputs. Furthermore, in order to examine differences in the aggregates nanostructure, a topographical evaluation of representative fibrillar structures was done, by performing several measurements of subsequent cylindrical segments along the fibril axis. The method described by the following equation (1) W* = W − 2√[H(2R_t_ – H)] was applied to the obtained width and height values in order to correct the geometry of the resulting convolved image [68]. In (1), W* and *W* represent the actual and observed fibril width from the AFM image, respectively, *H* is the height of the structure from the substrate and *R_t_* is the curvature radius of the tip (*R_t_* = 8 nm).

### Molecular Docking

Molecular docking was carried out using Autodock Vina program [69]. Structures of the carnosine or canosine-like dipeptides and the natural or synthetic β-amyloid aggregation inhibitors were obtained from the PubChem Compound database (**Table S1**) and *pdbqt*-formatted using the Open Babel Package 2.1.1 [70]. The simple monomer of the β-fibril model of Aβ1-42 (PDB: 2BEG) [55] was used as the receptor for docking calculations. For all docking studies the size of grid box was set to 45 Å×21 Å×11 Å to encompass the entire surface of monomer fibril, while grid spacing was set to the default value (0.375 Å). Docking was carried out with an exhaustiveness value of 8 and a maximum output of 10 structures and the best bound conformation for each docking simulation was chosen based on the lowest Autodock Vina predicted binding energy calculated in kcal/mol. Molecular graphics and analysis of docking results were performed using the UCSF Chimera package [71] (http://www.cgl.ucsf.edu/chimera).

### Molecular Properties and Efficiency Indices Calculation

Molecular size descriptors [i.e.: molecular weight (MW), number of heavy atoms (NHA), number of carbons (NoC) and Wiener index (W, a topological index defined as the sum of the edges in the shortest paths between all the heavy atoms)] of ligands were calculated using the Marvin Calculator Plugin (Version 5.10.3; http://www.chemaxon.com). The ligand efficiency indices were calculated as previously described [72], [73] by normalizing the Autodock Vina predicted free energy of binding of the ligand with respect to the different size descriptors.

## Results

### Effect of Carnosine on Aβ1-42 Fibrillogenesis

**Figure 1:**
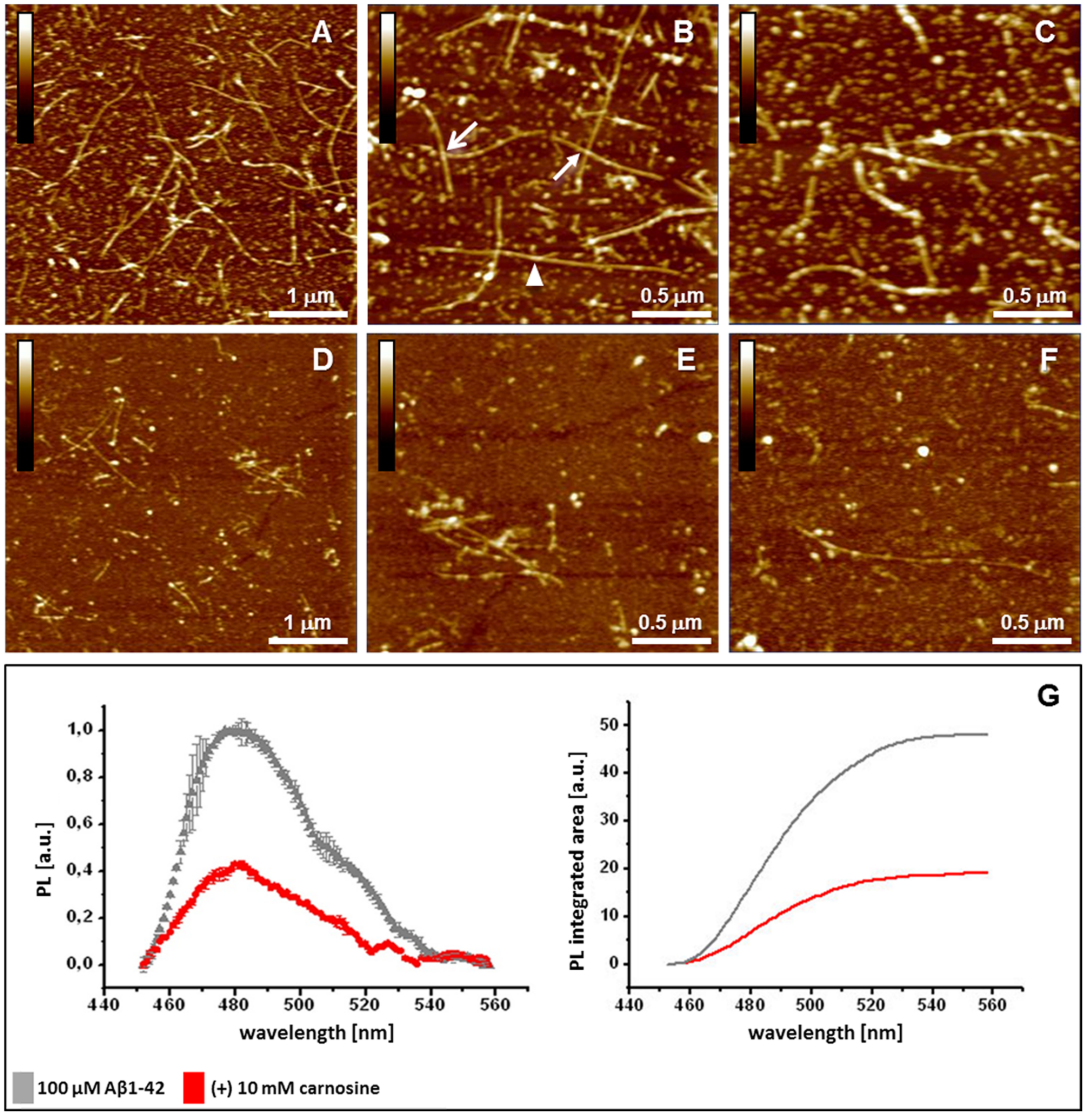
Effects of carnosine on Aβ1-42 fibrillogenesis. Analysis of the deposited amyloid aggregates as assessed by Atomic Force Microscopy (AFM) and thioflavin T (ThT) assays. AFM pictures (**A**–**F**) represent a comparative view of deposited Aβ1-42 amyloid aggregates, with representative fibrils from (**A–C**) Aβ1-42 samples (control) and (**D–F**) Aβ1-42/carnosine co-incubated samples. (**B, C**) Higher resolution images of (**A**) are reported, showing (**B**) extended structures of linear branched (arrowhead), overlapped (closed arrow) or associated (open arrow) fibrils; small fibrillar formations and oligomers among globular particles are also observed. (**E, F**) Higher resolution images of (**D**) are reported, showing spare fibrils and aggregates with respect to what observed in Aβ1-42 samples; conspicuous fibril segmentation and size reduction were observed [*Height* mode imaging; *Pico Force* type scanner; scanned area size: 5×5 µm in (**A**) and (**D**) and 2.5×2.5 µm in the others; height bars colour code: 0.0 nm, total black, 15 nm, total white]. (**G**) Quantitative effects of carnosine on Aβ1-42 fibrillogenesis by ThT assay. Data are expressed as ThT photoluminescence (PL; Y axis) values (means ± SD, n = 3) in solutions of Aβ1-42 (100 µM) incubated for 30 min in the absence (control) and presence of carnosine (10 mM). In the left graph, the maximum photoluminescence intensity (near wavelength 480 nm; X axis) is reduced in the co-incubated samples (red circles) with respect to the samples containing Aβ1-42 alone (grey triangles), passing from 1.0 to ∼ 0.4 absorbance units (a.u.). The emission spectrum of carnosine alone was subtracted, and emission data of peptide dispersions were normalized. In the graph on the right: fluorescence signals expressed as the integrated areas under the curves (OriginPro 8).

**Figure 2:**
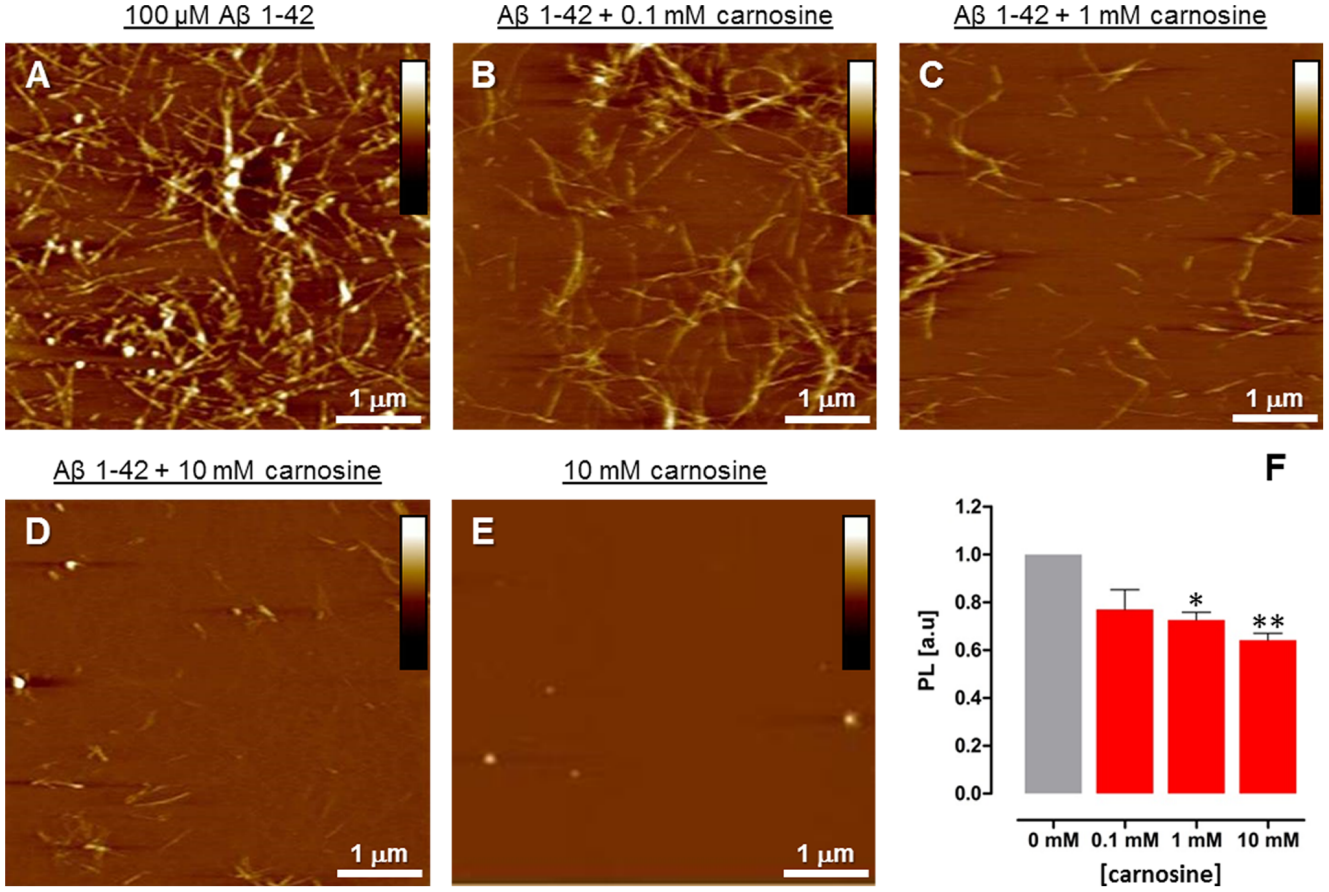
Dose-dependent effects of carnosine on Aβ1-42 fibrillogenesis. Analysis of the deposited amyloid aggregates as assessed by Atomic Force Microscopy (AFM) and thioflavin T (ThT) assays. AFM pictures (A–D) represent a view of deposited Aβ1-42 amyloid aggregates, with representative fibrils from Aβ1-42 samples (A; control) and Aβ1-42 samples incubated with 0.1 mM (B), 1 mM (C) and 10 mM (D) carnosine. AFM picture (E) of 10 mM carnosine alone. [Height mode imaging; Pico Force type scanner; scanned area size: 5×5 µm; height bars colour code: 0.0 nm, total black, 30 nm, total white]. (F) Quantitative effects of increasing concentrations of carnosine on Aβ1-42 fibrillogenesis by ThT assay. Data are represented as ThT photoluminescence (PL) values (means ± S.E.M., n = 3) in solutions of Aβ1-42 (100 µM) incubated for 30 min in the absence (control, 0 mM carnosine) and presence of 0.1, 1 and 10 mM carnosine. The photoluminescence intensity at 480 nm is reduced in the co-incubated samples in a dose-dependent manner with respect to the samples containing Aβ1-42 alone, passing from 1.0 to 0.64±0.03 absorbance units (a.u.). The emission value of carnosine alone was subtracted, and data were normalized with respect to the control (Aβ1-42 alone, 0 mM carnosine). (** p<0.01; * p<0.05; one-way ANOVA analysis of variance of the means; Bonferroni *post-hoc* test).

AFM imaging was used to evaluate fibrillogenesis of Aβ1-42 (100 µM) at physiological pH (7.4) in the absence and presence of excess carnosine (10 mM). After 30 min incubation at 37°C, imaging of the amyloid samples deposited on mica revealed the presence of both abundant and extended fibrillar structures and smaller aggregates (see **Fig. 1A–C**). Specifically, abundant linear fibrils were detected, with associated or overlapping filaments and branched-like structures, as well as smaller fibrillar formations, oligomers (≤0.1 µm) and globular particles. Conversely, co-incubation of Aβ1-42 with carnosine led to a different picture, with detection of spare fibrils, shorter than those observed in Aβ1-42 control samples, in addition to globular particles (**Fig. 1D–F**). Under the same fibrillogenesis conditions, ThT fluorescence assays confirmed that Aβ1-42 polymerization of amyloid aggregates was quantitatively reduced in the presence of carnosine 10 mM with respect to what observed in the absence of carnosine (**Fig. 1G**); such effect of carnosine was dose-dependent, as assessed in the 0–10 mM range (**Fig. 2**). Overall, in our experimental conditions the highest carnosine concentration (10 mM) lowered formation of Aβ1-42 aggregates from 40% to 60%, depending on the commercial lot of Aβ1-42 used. The observed inhibitory effect on the Aβ1-42 fibrillogenesis was due to carnosine and not to its component amino acids (**Fig. S1**). Anyhow, no carnosine hydrolysis occurred under the experimental conditions used for the fibrillogenesis assays, as assessed by HPLC analysis (see **Fig. S2**). Taken together, these results highlight the potential anti-aggregating effect of carnosine on Aβ1-42 polymerization and amyloid fibril formation *in vitro*.

### Effect of Carnosine on Aβ1-42 Fibril Morphology

**Figure 3:**
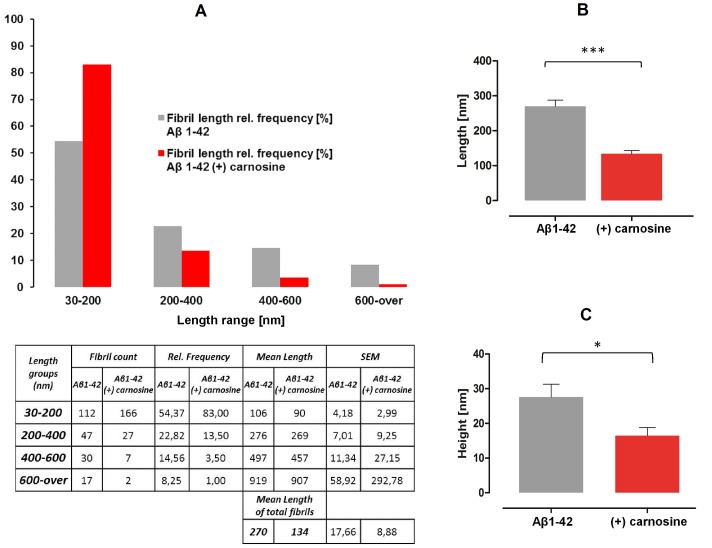
Effects of carnosine on Aβ1-42 fibril morphology: length and height analysis of deposited aggregates. (**A**) The fibril contour length distributions were calculated based on Atomic Force Microscopy measurements (grey and red bars refer to absence and presence of carnosine, respectively). Fibril sizes are grouped by length range (nm), while length distributions are reported as relative (%) frequency groups of the total number of measurements (n = 206 for Aβ1-42 and n = 202 for carnosine co-incubated samples; see table related to figure A for statistical details). (**B**) The mean values of the total fibril length measurements are reported for fibrils detected in the absence (grey bars) or presence (red bars) of carnosine (*** p<0.0001; unpaired *t* test). (**C**) Height digital data were obtained by scanning areas of 5×5 µm from Aβ1-42 alone (grey bars) and carnosine co-incubated samples (red bars), using the Nanoscope Software 7.3 roughness routine (* p<0.05; n = 5; unpaired *t* test).

Incubation of Aβ1-42 (100 µM) with carnosine (10 mM) affected fibril morphology in terms of decrease of frequency of longer Aβ1-42 fibril formations, and increase of shorter. To get relative quantitation of frequency, the fibril contour length distributions were calculated for fibrils deposited both in the absence and presence of carnosine. Measurements revealed that interaction with carnosine strongly reduced the frequency of Aβ1-42 fibrils longer than 200 nm, and increased the frequency of protofibrils or short fibrils in the range 30–200 nm, with respect to control (**Fig. 3A**). Overall, the mean values of fibril length of deposited amyloid aggregates was reduced from 270±17.7 nm in the absence of carnosine to 134±8.9 nm when carnosine was co-incubated (**Fig. 3B**). By further processing of the acquired AFM images, the output values of the analysis of the surface roughness profiles from the mean data plane were obtained to evaluate the mean peak height of the amyloid formations deposited in Aβ1-42 samples and in Aβ1-42 co-incubated with carnosine. A reduction of the mean peak height of the deposited aggregates from 27.6±3.69 to 16.5±2.31 nm was observed, passing from Aβ1-42 alone to carnosine co-incubated samples (**Fig. 3C**).

**Figure 4:**
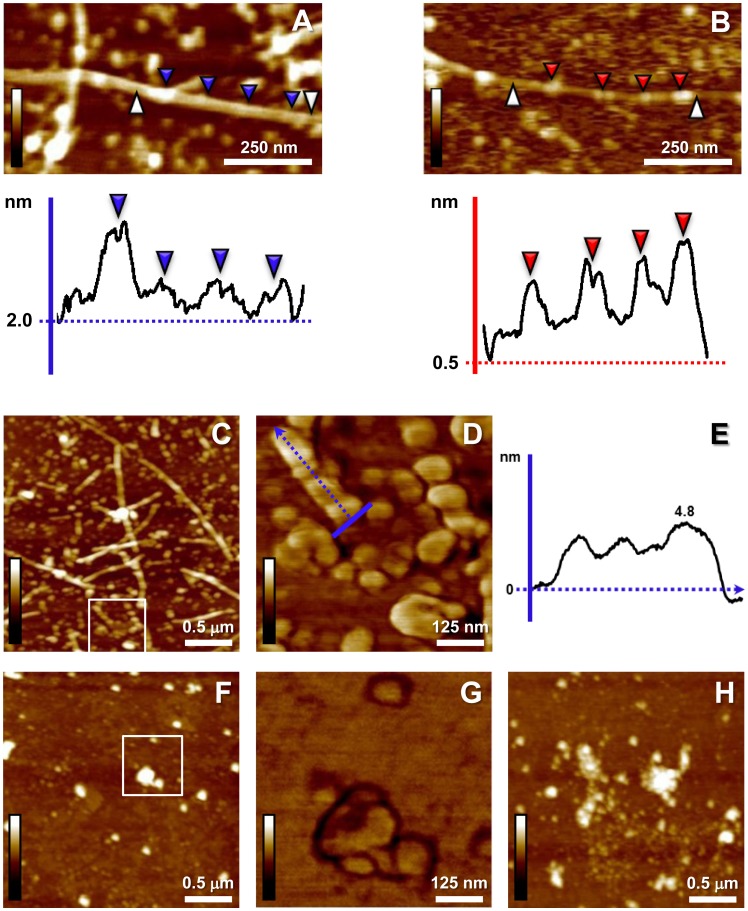
Effects of carnosine on Aβ1-42 fibrillogenesis: structural changes of fibrils and morphology of sub-fibrillar aggregates. (**A**) Magnification of a representative fibril from a control sample (from **Figure 1B**; 100 µM Aβ1-42). The related graphic panel reports the surface profile of a selected segment (white arrowheads, from left to right). A regular structure periodicity is shown, with relative height homogeneity among different pointed regions (blue arrowheads), except in protrusion (or interacting) sites (first blue arrowhead, left); for the fibril surface structure, a baseline height of 2 nm from substrate level is reported. (**B**) The profile of the selected segment (white arrowheads, from left to right) from a digitally zoomed sporadic fibril from carnosine (10 mM) co-incubated samples shows tangled pattern, irregular vertical height from the baseline (0.5 nm), and alternation between beaded regions (red arrowheads) and tubular segments (height bars colour code in **A**, **B**: 0.0 nm, total black, 10.0 nm, total white; *Height* mode imaging; Nanoscope 7.3 Section Analysis tool with no flatten filter applied). (**C**) Higher resolution scansion of Aβ1-42 fibrils in the absence of carnosine: aggregates of reduced size (protofibrillar/oligomeric formations, globular particles) are detected among fibrils. (**D**) Higher magnification (*Phase* signal; white square inset from **C**) shows pre-fibrillar organization of the amyloid structures as ordered rows with constant and homogeneous topographic profile along the axis (blue arrow in **D** indicates the profile direction reported in **E**). (**F**) Magnifications of carnosine co-incubated samples do not show similar ordered patterns of the sub-fibrillar dispersed aggregates (**G**, *Phase* mode imaging, white square inset from **F**); aggregates show size heterogeneity and less regular shape. (**H**) Quasi-spherical and decorated aggregates are typically visible in the carnosine co-incubation. Imaging from **C** to **H** performed with *E* type scanner; specific scan sizes: 2.5×2.5 µm in **C**, **F**, **H** and 0.625×0.625 µm in **D**, **G**; height bars colour code: 0.0 nm, total black, 15.0 nm, total white.

Deeper morphological analysis was conducted on single fibril digital magnifications to reveal qualitative shape-contour differences between deposited preparations in the absence or presence of carnosine. When the height profiles were analyzed, fibrils from control samples exhibited a likely constant structure periodicity and height variation was consistent with branching or overlapping structures sites (**Fig. 4A**). On the other hand, analyzing surface profiles of sporadic fibrils from carnosine co-incubated samples a less constant structure periodicity along the filament, and lower baseline level than that detected in the control samples were recorded (**Fig. 4B**); specifically, marked alternation of both beaded and tubular segments could be noticed more often along fibrils in the presence of carnosine, thus corresponding to minor homogeneity of the deposited structures. Consistently, the average thickness along these representative fibrillar structures seemed reduced in carnosine co-incubated samples (from 24.87 to 17.96 nm in the carnosine co-incubated sample; deconvoluted value of representative data). More clearly, higher resolution AFM scansion (*E* type scanner) of Aβ1-42 amyloid fibrils in the absence of carnosine showed deposition of aggregates of reduced size, such as small protofibrillar, oligomeric formations, and globular particles, within a network of extended fibrils (**Fig. 4C**); imaging of the aggregates revealed possible pre-fibrillar organization of the amyloid structures, such as ordered rows retaining a constant and homogeneous height profile, composed of closely spaced/linked beads of comparable size (**Fig. 4D, E**). On the other hand, when carnosine was co-incubated, sub-fibrillar Aβ1-42 aggregates detected did not show any similar structural organization or possible ordered patterns, appearing dispersed and showing size heterogeneity (**Fig. 4F, G**); quasi-spherical and decorated aggregates were also typically detectable only in Aβ1-42 amyloid deposits from carnosine co-incubated samples (**Fig. 4H**). Overall, the quantitative analysis of the fibril length distributions together with the in-depth AFM imaging of the deposited aggregates confirmed the evidence that less Aβ1-42 amyloid aggregation occurred, with less fibril growth, in the presence of carnosine, assessing structural and morphological rearrangements due to the dipeptide action leading to abortive dynamics of amyloid fibrillogenesis by Aβ1-42.

### Molecular Docking Analysis of Aβ1-42 Aggregation Inhibition by Carnosine

**Figure 5:**
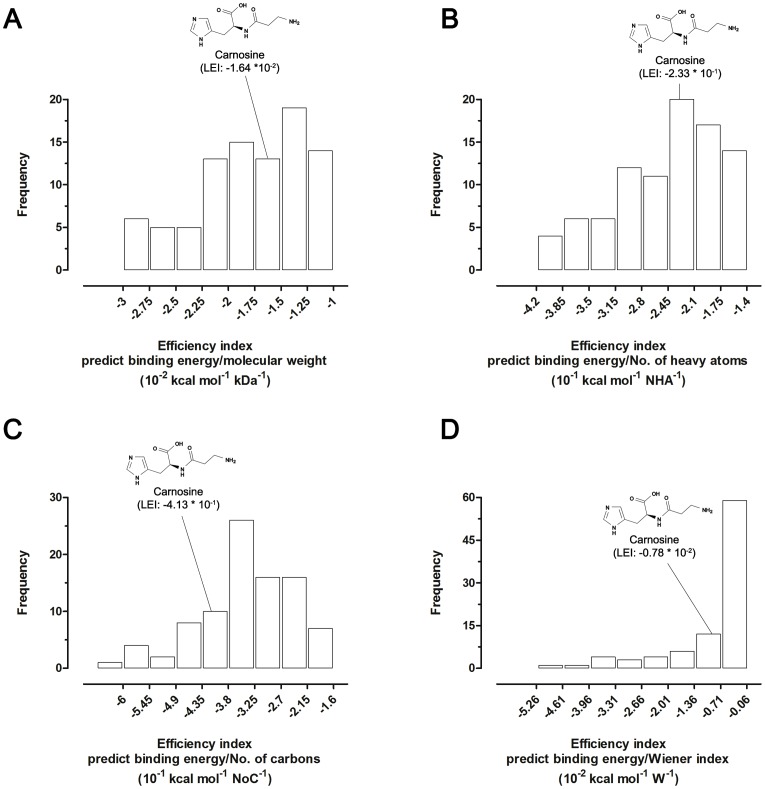
Frequency distributions of ligand efficiency indices (LEIs). LEIs were obtained by using the Autodock Vina predicted binding free energies calculated for carnosine, carnosine-like dipeptides and natural or synthetic anti-amyloid aggregation compounds *vs* Aβ1-42. Relative positions of the carnosine score in the distribution graphs were indicated. The same number of bins were applied for all the histograms. (**A**) Molecular weight-based efficiency index (free energy of binding/MW). (**B**) Number of heavy atoms-based efficiency index (free energy of binding/NHA). (**C**) Number of carbons-based efficiency index (free energy of binding/NoC). (**D**) Wiener index-based efficiency index (free energy of binding/W).

To characterize the molecular mechanisms/interactions by which carnosine can inhibit Aβ1-42 aggregation, a molecular docking analysis was employed. Docking calculation of a data set of 89 compounds, composed of three different carnosine-like dipeptides (homocarnosine, anserine and balenine) and 86 selected inhibitors of the Aβ1-42 aggregation, was performed in the same analysis to compare docking results and to explore variations on ligand efficiencies. All compounds were ranked according to four ligand efficiency indices and frequency distribution graphs were composed accordingly (**Fig. 5**). The *in silico* screening of Aβ1-42 ligands predicted carnosine as the best ligand among the natural histidine-containing dipeptides tested (**Table S1**), and as a very good ligand when compared with other compounds capable of inhibiting the aggregation of the Aβ1-42 amyloid peptide (**Fig. 5** **and Table S1**).

**Figure 6:**
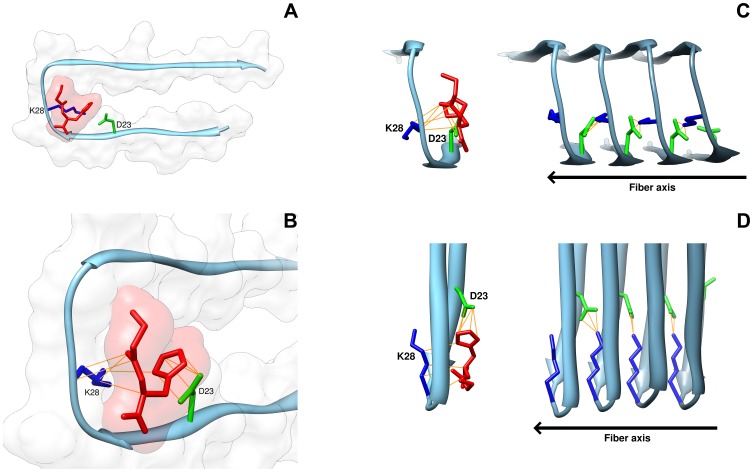
Three-dimensional model of interactions of carnosine with the fibril of the Aβ1-42 peptide. Binding mode of carnosine with the fibril structure of Aβ1-42 (PDB Acc. no. 2BEG) was obtained using Autodock Vina and visualized by UCSF Chimera software. (**A**) The carnosine dipeptide interacts with the fibril monomer of Aβ1-42 at the level of the coiled region between the two β-sheet portions of the Aβ1-42 peptide. The fibril monomer is represented by tube, and the secondary structure is reported colored in sky-blue; the dipeptide is depicted by tube colored in red. (**B**) Direct binding contacts (yellow lines) occur between the imidazole ring of L-histidine in carnosine and residue D23 (green) of Aβ1-42, and between the β-alanine end of carnosine and amino acid K28 (blue) of Aβ1-42. Surface renditions of the binding interface of carnosine and amyloid peptide are shown. (**C**) The self-association process of Aβ1-42 is inhibited by carnosine. The interaction of carnosine with the Aβ1-42 monomer prevents the direct binding of the D23 of a monomer with the K28 of the adjacent monomer in the growing Aβ1-42 oligomer. The elongation/growth direction of Aβ1-42 aggregation is reported as fiber axis. (**D**) A 90° clockwise rotation view (around the fiber axis) of the of Aβ1-42 self-association model inhibited by carnosine.

Docking simulations placed carnosine at the level of the central coiled region of the Aβ1-42 peptide (**Fig. 6**). In particular, the predicted binding mode for carnosine and Aβ1-42 fibril displayed a close interactions between the natural dipeptide and the residues D23 (L-aspartic) and K28 (L-lysine) of Aβ1-42 (**Fig. 6A**), with direct contacts occurring between the D23 residue of Aβ1-42 and the imidazole ring of carnosine, and between the K28 residue of Aβ1-42 and the β-alanine end of dipeptide (**Fig. 6B**). This proposed pose was in agreement with the anti-aggregating properties observed *in vitro* for carnosine, being the D23 and K28 residues directly involved in the Aβ1-42 self-association process by forming an intermolecular salt bridge between two adjacent Aβ1-42 monomers [55]. In this context, the binding of carnosine to the Aβ1-42 monomer could prevent the intermolecular salt bridge formation, thus inhibiting the fibril aggregation process (**Fig. 6C, D**).

Overall, molecular docking results supported the notion that carnosine efficiently binds to the Aβ1-42 peptide and the evidence that impairs fibril formation *in vitro*.

## Discussion

Due to the high levels of natural synthesis occurring in several nervous cell types, carnosine is a dipeptide endogenously abundant in many CNS districts. Its potential in counteracting neurodegenerative effects arising from altered protein accumulation and toxicity has been studied, and its protective effects against aberrant amyloid peptides have been investigated in mammalian tissues and cells [28], [30], [31]. Nevertheless, the direct impact of carnosine on the dynamics of the AD-related Aβ1-42 fibril formation remains completely uninvestigated. Thus, we analyzed *in vitro* the effects of carnosine on Aβ1-42 fibrillogenesis, which was carried out at physiological pH (7.4), temperature (37°C) and carnosine levels (0.1–10 mM), based on data from mammalian nervous tissues [74], [75] as well as on our experimental measurements (data not shown). AFM was used as method of choice since it successfully reveals basic and hierarchical aspects of amyloid fibril structure formation [62], [63], [76], with ThT assay supporting AFM to quantitatively examine the alterations of self-assembled Aβ1-42 amyloid aggregates.

As also described by others [62], [63], [76], our AFM images of deposited Aβ1-42 reveal extended linear and branched fibrils (>1 µm) and smaller structures resembling in size protofibrillar and oligomeric formations, among many globular aggregates. Conversely, in the presence of carnosine extended fibrils appear drastically underrepresented, while the few deposited aggregates predominantly include shorter fibrils and small globular formations. This evidence of reduced Aβ1-42 aggregation is confirmed quantitatively by ThT fluorescence assay, showing a reduction of the Aβ1-42 polymerization process in the presence of increasing concentrations of carnosine. Moreover, the overall count of the deposited aggregates was shown to be significantly decreased in a dose-dependent manner (**Fig. S3**), suggesting that disrupting or disaggregating effects (probably leading to a number of deposited aggregates greater than or equal to the control) should not even occur. It has to be noticed that in our experiments carnosine effects were evaluated after 30 min incubations, that is a time interval referred to very early phases of Aβ1-42 amyloid fibrillogenesis *in vitro*, during which carnosine could interact/interfere mainly with growing oligomeric aggregates and protofibrils [43], [63]. Taken together, our results indicate an important anti-aggregating effect of carnosine on early Aβ1-42 polymerization and amyloid fibril formation *in vitro*.

Besides the evidence to reduce the total number of aggregates, carnosine specifically affects the frequency of extended Aβ1-42 fibril formations, and short-sized fibrillar aggregates prevail in the presence of the dipeptide. The analysis of the fibril contour length distribution clearly indicates that carnosine/Aβ1-42 interaction leads to increased relative frequency of short fibrillar aggregates from 30 to 200 nm, that is the range corresponding to lengths reported for small protofibrils or premature fibrils [43], [55]. On the other hand, frequency of Aβ1-42 fibrils longer than 200 nm is largely reduced by carnosine. Overall, the mean fibril lengths of deposited aggregates appear almost halved. Moreover, the evidence of a reduction of the mean peak height of the deposited aggregates in the presence of carnosine supports the idea that carnosine interaction does have an impact on the structural morphology of the growing fibrillar aggregates. On these bases, deeper morphological analysis was conducted on single fibrils to reveal qualitative differences between amyloid structures deposited in the absence and presence of carnosine. Analyzing the height profiles, fibrils from control samples exhibit structural homogeneity, whereas the sporadic fibrils from carnosine-treated samples show less regular periodicity along the surface profile and more irregular vertical distances from the substrate baseline, with evident alternation of both beaded and tubular segments along fibrils. These findings indicate minor structural homogeneity of the aggregated fibrils (as well as irregular polymerization) with respect to untreated Aβ1-42 samples. In addition, besides the dense network of extended fibrils, Aβ1-42 samples incubated in the absence of carnosine show very small fibril-like or globular formations, resembling in size (<30 nm) the soluble oligomeric species reported frequently in structure-function studies of Aβ1-42 amyloid fibrils in AD performed by using AFM imaging and 3D structure modeling [43], [55], [77]. Detailed imaging of these aggregates reveals pre-fibrillar organization of the amyloid structures, such as ordered rows of linked beads with homogeneous height profiles. Carnosine modifies such a kind of sub-fibrillar Aβ1-42 aggregation states. In fact, the deposited formations detected in the presence of the dipeptide do not show any structured organization or ordered patterns such as those detected in its absence, and invariably appear heterogeneous in size and shape. Overall, the analysis of the fibril length, together with the detailed morphological analysis of shapes and structures of the deposited aggregates, suggest that carnosine induces a less ordered Aβ1-42 amyloid aggregation, with less fibril growth. This suggests structural and morphological rearrangements due to carnosine inhibitory effects on Aβ1-42 fibrillogenesis in its early phases.

For what discussed above, carnosine seems to operate as an interfering, anti-aggregating agent, with evident effects on Aβ1-42 small pre-fibrillar structures. Thus, a molecular docking approach was exploited to specifically predict the molecular interactions between carnosine and the Aβ1-42 peptide and to evaluate the anti-aggregating behavior of carnosine. Ligand efficiency indices, calculated for carnosine and for a large data set of compounds exerting anti-aggregating effects against Aβ1-42 fibrillogenesis, were used as tools to predict and “measure” the drug-likeness of compounds [78], [79]. Coherently with the experimental evidence, *in silico* analysis recognized carnosine as a potential drug candidate, predicting ligand efficiency indices equal to or better (i.e. deeper) than values reported in previous drug screening studies [80], [81] (**Table S1**). Moreover, the extensive molecular docking studies performed on several known inhibitors of the Aβ1-42 fibril formation, groups carnosine together with the top-ranked compounds (**Fig. 4** and **Table S1)**, confirming carnosine as a good inhibitor of the fibril aggregation process.

The binding mode of carnosine on the Aβ1-42 shows that the dipeptide interacts with a coiled region between the two β-sheet portions of the Aβ1-42 peptide in the folded conformational state, that is the amyloid peptide conformation occurring during the amyloid fibril polymerization process [55]. In detail, the analysis shows arrangement of carnosine in a region where two amino acid residues, namely D23 and K28, are present. Such residues are known to be crucial in the intermolecular interactions between two adjacent Aβ1-42 monomers during the elongation of protofibrils, according to the model of growth by single monomer addition [55]. Docking clearly reveals oriented contacts between the imidazole ring of the L-histidine of carnosine and the D23 residue of Aβ1-42, and between the β-alanine of the dipeptide and the K28 residue of Aβ1-42. Docking of carnosine on Aβ1-42 in the oligomeric/fibrillar form reasonably confirms the potential interference of carnosine on fibrillogenesis, and its ability to unsettle the growing fibril by impeding direct binding of the D23 of one Aβ1-42 monomer and the K28 of the adjacent monomer. Overall, our docking analysis suggests that relevant molecular interactions may occur between carnosine and Aβ1-42, thus disrupting the organization of the growing small protofibrils and the self-association process, according to our experimental results of impaired fibril formation *in vitro*.

### Conclusions

In summary, carnosine appears to operate as a relevant interfering, anti-aggregating agent against Aβ1-42 small pre-fibrillar structures. To date, small oligomers are considered the major aggressive variant of the amyloid formations [43], and the so-called “oligomer cascade hypothesis” is becoming a key premise in studies concerning the structure-neurotoxicity relationships of the amyloid formations [82]. Our results give hints for disclosing the crucial role of carnosine and its homeostasis in the context of Aβ1-42 amyloid fibril formation, a topic that needs further investigation in consideration of carnosine pathophysiological potential deriving from its natural occurrence in the CNS.

## Supporting Information

Figure S1 **Effect of hydrolysed carnosine (β-alanine and L-histidine) on Aβ1-42 fibrillogenesis.** Analysis of the deposited amyloid aggregates as assessed by Atomic Force Microscopy (AFM) and thioflavin T (ThT) assays. AFM pictures represent a view of deposited Aβ1-42 amyloid aggregates, with representative fibrils from Aβ1-42 samples (0 mM carnosine; control) and Aβ1-42 samples incubated with 10 mM hydrolysed carnosine (β-alanine and L-histidine, 10 mM each). [Height mode imaging; Pico Force type scanner; scanned area size: 5×5 µm; height bars colour code: 0.0 nm, total black, 30 nm, total white]. The graphic below shows quantitative effects of hydrolised carnosine on Aβ1-42 fibrillogenesis by ThT assay. Data are represented as ThT photoluminescence (PL) values (means ± S.E.M., n = 3) in solutions of Aβ1-42 (100 µM) incubated for 30 min in the absence (control, 0 mM carnosine) and presence of 10 mM hydrolised carnosine. The 480 nm photoluminescence intensity mean values were not statistically different (*t test* analysis of the means). Photoluminescence appears only faintly reduced in the co-incubated samples with respect to the samples containing Aβ1-42 alone, passing from 34700±2764 (100%) to 30100±2541 (87%) absorbance units (a.u.). The emission value of carnosine alone was subtracted.
(TIF)

Figure S2 **Reverse Phase High Performance Liquid Chromatography (RP-HPLC) for detection of carnosine hydrolysis under the fibrillogenesis buffering conditions.** Solutions of carnosine (10 mM), L-histidine (10 mM), and carnosine (5 mM) plus L-histidine (5 mM) in Tris-HCl buffer (50 mM, pH 7.4), were incubated 30 min at 37°C and then immediately processed by RP-HPLC. To evaluate hydrolysis of carnosine, possibly due to the buffer solution used to perform the fibrillogenesis assays (see previous sections), solutions of 10 mM carnosine, 10 mM L-histidine, and 5 mM carnosine plus 5 mM L-histidine in 50 mM Tris-HCl at pH 7.4 were incubated for 30 min at 37°C and subsequently injected for RP-HPLC analysis. A Hewlett-Packard 1100 Series isocratic system equipped with a variable wavelength detector was used, and the RP-HPLC conditions adopted were as follows: Hypersil column ODS 4.6×250 mm, 5 µm (particle size); column temperature 40°C; isocratic elution with 0.1% (v/v) trifluoroacetic acid (TFA) in 95∶5 water:acetonitrile; flux 1 mL/min; UV absorbance at 214 nm. Peaks with different retention times were detected for carnosine and L-histidine (3.7 min and 3.34 min, respectively), as well as for the equimolar mix of carnosine and L-histidine. In particular, no L-histidine peak, due to the possible hydrolysis of the dipeptide, was detected in the carnosine sample. No peaks were detected with the buffer alone (Tris-HCl 50 mM, pH 7.4). X axis: retention time (min); Y axis: Absorbance Units (mAU) (at 214 nm).
(TIF)

Figure S3 **Dose-dependent effects of carnosine on the number of deposited aggregates.** Analysis of the number of deposited amyloid aggregates as assessed by Atomic Force Microscopy (AFM) images. AFM pictures (A–D) represent a view of deposited Aβ1-42 amyloid aggregates, with representative fibrils from Aβ1-42 samples (A; control) and Aβ1-42 samples incubated with 0.1 mM (B), 1 mM (C) and 10 mM (D) carnosine [Height mode imaging; Pico Force type scanner; scanned area size: 5×5 µm; height bars colour code: 0.0 nm, total black, 30 nm, total white]. Data are represented as counts of the detected aggregates deposited on mica (means ± S.E.M., n = 4) in different samples of Aβ1-42 (100 µM) incubated for 30 min in the absence (control, 0 mM carnosine) and presence of 0.1, 1 and 10 mM carnosine (*** p<0.001; one-way ANOVA analysis of variance of the means; Bonferroni *post-hoc* test).
(TIF)

Table S1 **Ligand efficiency indices.** Ligand efficiency indices calculated from molecular docking analysis of the β-fibril model of Aβ1-42 (PDB: 2BEG) *vs* carnosine and 89 selected molecules, including: a) carnosine-like dipeptides; b) natural or synthetic compounds tested for anti-amyloid aggregation effects. Ligand efficiency indices are indicated as BE/MW (molecular weight-based efficiency index), BE/NHA (number of heavy atoms-based efficiency index) BE/NoC (number of carbons-based efficiency index) BE/W (Wiener index-based efficiency index).
(DOC)
