# Supplementary figures and images for: Anti-Aggregating Effect of the Naturally Occurring Dipeptide Carnosine on Aβ1-42 Fibril Formation
Source: PLoS One. 2013 Jul 3;8(7):e68159. doi: 10.1371/journal.pone.0068159 (PMC3700870; doi:10.1371/journal.pone.0068159)

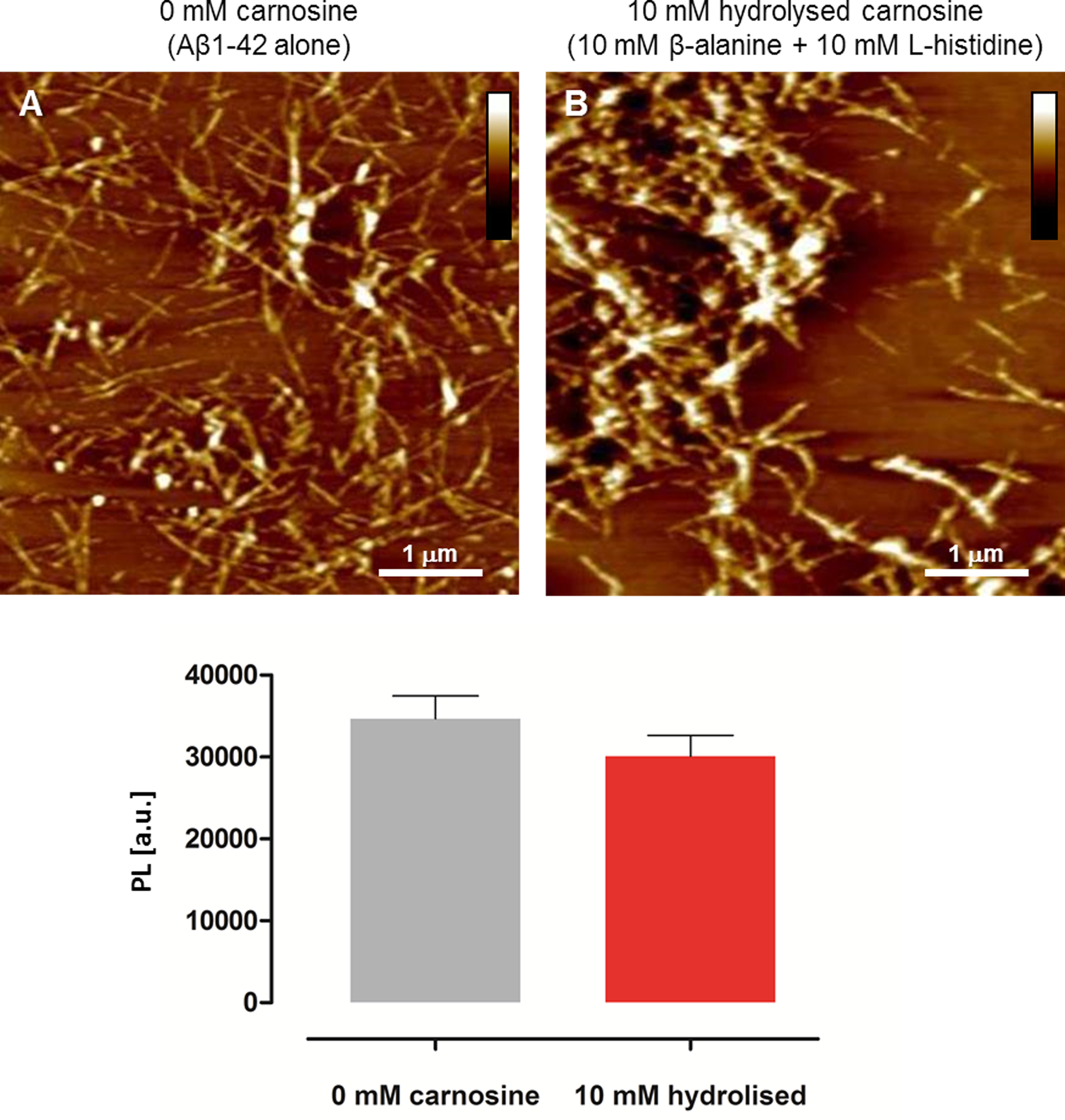

Supplement: Figure S1 — Effect of hydrolysed carnosine (β-alanine and L-histidine) on Aβ1-42 fibrillogenesis. Analysis of the deposited amyloid aggregates as assessed by Atomic Force Microscopy (AFM) and thioflavin T (ThT) assays. AFM pictures represent a view of deposited Aβ1-42 amyloid aggregates, with representative fibrils from Aβ1-42 samples (0 mM carnosine; control) and Aβ1-42 samples incubated with 10 mM hydrolysed carnosine (β-alanine and L-histidine, 10 mM each). [Height mode imaging; Pico Force type scanner; scanned area size: 5×5 µm; height bars colour code: 0.0 nm, total black, 30 nm, total white]. The graphic below shows quantitative effects of hydrolised carnosine on Aβ1-42 fibrillogenesis by ThT assay. Data are represented as ThT photoluminescence (PL) values (means ± S.E.M., n = 3) in solutions of Aβ1-42 (100 µM) incubated for 30 min in the absence (control, 0 mM carnosine) and presence of 10 mM hydrolised carnosine. The 480 nm photoluminescence intensity mean values were not statistically different (t test analysis of the means). Photoluminescence appears only faintly reduced in the co-incubated samples with respect to the samples containing Aβ1-42 alone, passing from 34700±2764 (100%) to 30100±2541 (87%) absorbance units (a.u.). The emission value of carnosine alone was subtracted. (TIF) [file pone.0068159.s001.tif]

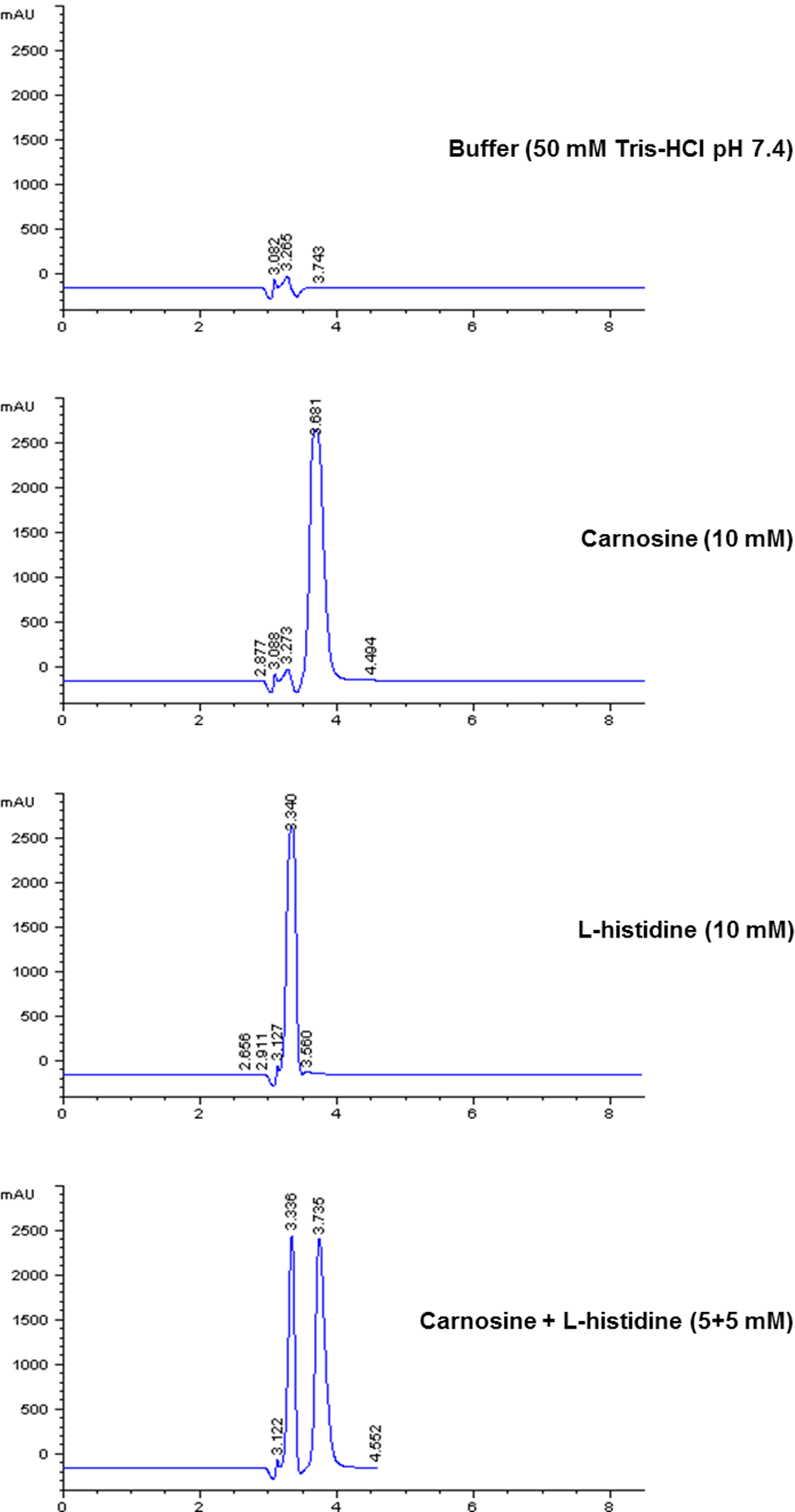

Supplement: Figure S2 — Reverse Phase High Performance Liquid Chromatography (RP-HPLC) for detection of carnosine hydrolysis under the fibrillogenesis buffering conditions. Solutions of carnosine (10 mM), L-histidine (10 mM), and carnosine (5 mM) plus L-histidine (5 mM) in Tris-HCl buffer (50 mM, pH 7.4), were incubated 30 min at 37°C and then immediately processed by RP-HPLC. To evaluate hydrolysis of carnosine, possibly due to the buffer solution used to perform the fibrillogenesis assays (see previous sections), solutions of 10 mM carnosine, 10 mM L-histidine, and 5 mM carnosine plus 5 mM L-histidine in 50 mM Tris-HCl at pH 7.4 were incubated for 30 min at 37°C and subsequently injected for RP-HPLC analysis. A Hewlett-Packard 1100 Series isocratic system equipped with a variable wavelength detector was used, and the RP-HPLC conditions adopted were as follows: Hypersil column ODS 4.6×250 mm, 5 µm (particle size); column temperature 40°C; isocratic elution with 0.1% (v/v) trifluoroacetic acid (TFA) in 95∶5 water:acetonitrile; flux 1 mL/min; UV absorbance at 214 nm. Peaks with different retention times were detected for carnosine and L-histidine (3.7 min and 3.34 min, respectively), as well as for the equimolar mix of carnosine and L-histidine. In particular, no L-histidine peak, due to the possible hydrolysis of the dipeptide, was detected in the carnosine sample. No peaks were detected with the buffer alone (Tris-HCl 50 mM, pH 7.4). X axis: retention time (min); Y axis: Absorbance Units (mAU) (at 214 nm). (TIF) [file pone.0068159.s002.tif]

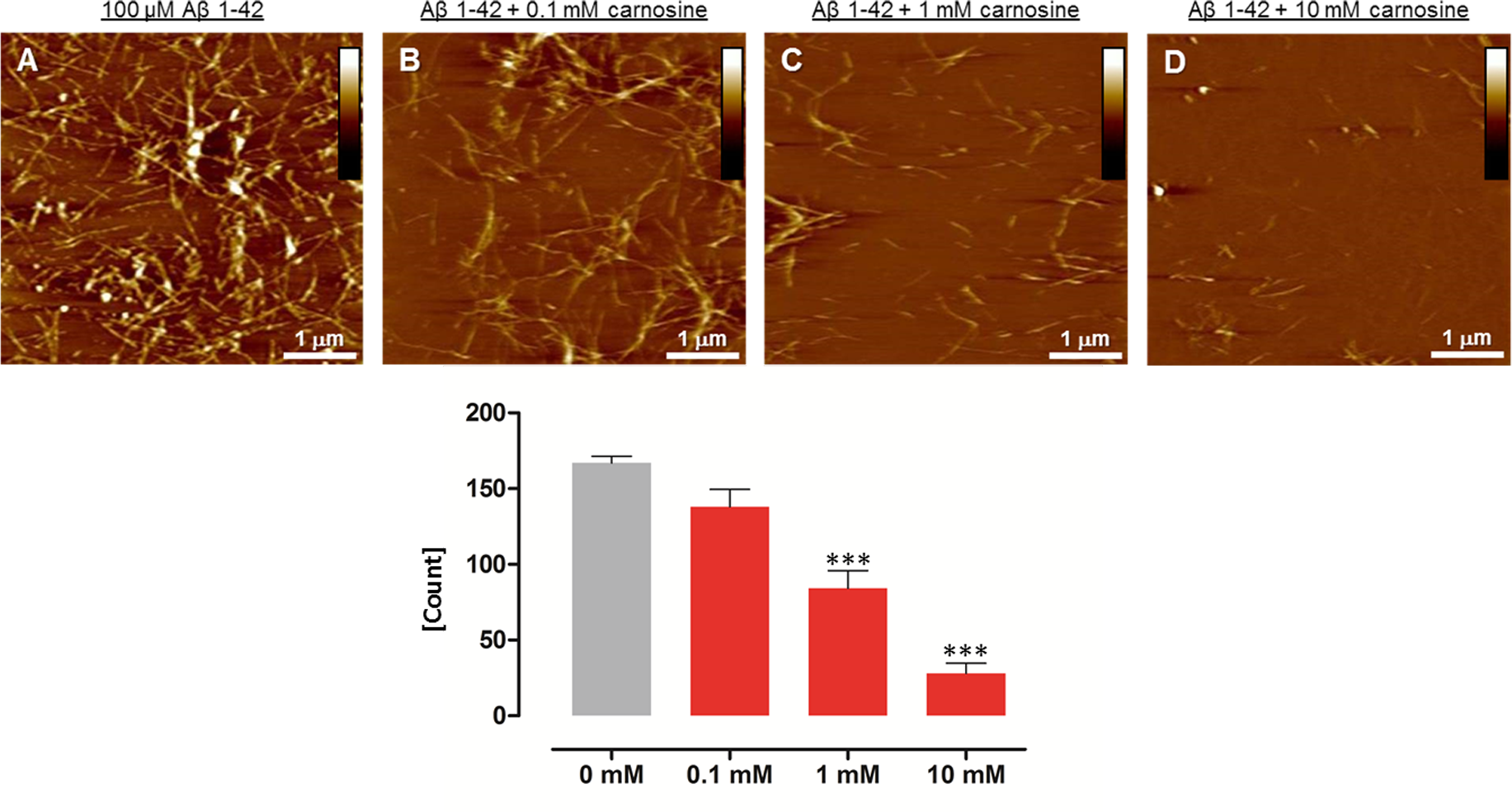

Supplement: Figure S3 — Dose-dependent effects of carnosine on the number of deposited aggregates. Analysis of the number of deposited amyloid aggregates as assessed by Atomic Force Microscopy (AFM) images. AFM pictures (A–D) represent a view of deposited Aβ1-42 amyloid aggregates, with representative fibrils from Aβ1-42 samples (A; control) and Aβ1-42 samples incubated with 0.1 mM (B), 1 mM (C) and 10 mM (D) carnosine [Height mode imaging; Pico Force type scanner; scanned area size: 5×5 µm; height bars colour code: 0.0 nm, total black, 30 nm, total white]. Data are represented as counts of the detected aggregates deposited on mica (means ± S.E.M., n = 4) in different samples of Aβ1-42 (100 µM) incubated for 30 min in the absence (control, 0 mM carnosine) and presence of 0.1, 1 and 10 mM carnosine (*** p<0.001; one-way ANOVA analysis of variance of the means; Bonferroni post-hoc test). (TIF) [file pone.0068159.s003.tif]
