# Supplementary material for: Anti-Aggregating Effect of the Naturally Occurring Dipeptide Carnosine on Aβ1-42 Fibril Formation
Source: PLoS One. 2013 Jul 3;8(7):e68159. doi: 10.1371/journal.pone.0068159 (PMC3700870; doi:10.1371/journal.pone.0068159)
Supplement: Table S1 — Ligand efficiency indices. Ligand efficiency indices calculated from molecular docking analysis of the β-fibril model of Aβ1-42 (PDB: 2BEG) vs carnosine and 89 selected molecules, including: a) carnosine-like dipeptides; b) natural or synthetic compounds tested for anti-amyloid aggregation effects. Ligand efficiency indices are indicated as BE/MW (molecular weight-based efficiency index), BE/NHA (number of heavy atoms-based efficiency index) BE/NoC (number of carbons-based efficiency index) BE/W (Wiener index-based efficiency index). (DOC) [file pone.0068159.s004.doc]

**Table S1.** **Ligand efficiency indices.** Ligand efficiency indices calculated from molecular docking analysis of the β-fibril model of Aβ1-42 (PDB: 2BEG) *vs* carnosine and 89 selected molecules, including: a) carnosine-like dipeptides; b) natural or synthetic compounds tested for anti-amyloid aggregation effects. Ligand efficiency indices are indicated as BE/MW (molecular weight-based efficiency index), BE/NHA (number of heavy atoms-based efficiency index) BE/NoC (number of carbons-based efficiency index) BE/W (Wiener index-based efficiency index).

| **#** | **Molecule name** | **PubChem ID** | **Ligand Efficiency Index** | | | | **References** |
| --- | --- | --- | --- | --- | --- | --- | --- |
|  |  |  | **BE/MW**  (10^-2^ kcal mol^-1^ kDa^-1^) | **BE/NHA**  (10^-1^ kcal mol^-1^ NHA^-1^) | **BE/NoC**  (10^-1^ kcal mol^-1^ NoC^-1^) | **BE/W**  (10^-2^ kcal mol^-1^ W^-1^) |  |
| **CARNOSINE AND CARNOSINE-LIKE DIPEPTIDES** | | | | | | | |
| 1 | Carnosine | 439224 | -1.64 | -2.33 | -4.13 | -0.78 | - |
| 2 | L-anserine | 112072 | -1.61 | -2.28 | -3.88 | -0.70 | - |
| 3 | Homocarnosine | 10243361 | -1.59 | -2.25 | -3.82 | -0.66 | - |
| 4 | Balenine | 3080547 | -1.44 | -2.04 | -3.46 | -0.58 | - |
| **NATURAL OR SYNTHETIC COMPOUNDS TESTED FOR ANTI-AMYLOID AGGREGATION EFFECTS** | | | | | | | |
| 5 | (3Z)-5,6-dihydroxy-1H-indole-2,3-dione-3-[(3-chlorophenyl)hydrazone] | 54732613 | -1.83 | -2.65 | -3.97 | -0.58 | [1] |
| 6 | (trans,trans)-1-bromo-2,5-bis-(3-hydroxycarbonyl-4-hydroxy)styrylbenzene (BSB) | 10184360 | -1.38 | -2.14 | -2.77 | -0.20 | [2] |
| 7 | 1,2-naphthoquinone | 10667 | -2.96 | -3.90 | -4.68 | -2.63 | [3] |
| 8 | 2-(4-(2-(3,4-dichlorophenyl)ethyl)phenylamino)benzoic acid | 9864959 | -1.62 | -2.41 | -2.98 | -0.31 | [4] |
| 9 | 2,2'-dihydroxybenzophenone | 70038 | -2.24 | -3.00 | -3.69 | -1.13 | [5] |
| 10 | 2-amino-4-chlorophenol | 7265 | -2.41 | -3.84 | -5.77 | -4.12 | [6] |
| 11 | 3-(4-naphthalen-1-yltriazol-1-yl)chromen-2-one | 11674496 | -2.13 | -2.78 | -3.45 | -0.43 | [7] |
| 12 | 3-(4-phenyltriazol-1-yl)chromen-2-one | 11687872 | -2.18 | -2.86 | -3.71 | -0.58 | [7] |
| 13 | 3-acetyl-7-(diethylamino)-2-oxochromene-4-carbonitrile | 13707550 | -1.78 | -2.41 | -3.16 | -0.57 | [7] |
| 14 | 3-hydroxy-4-phenoxybenzoic acid | 22133420 | -2.06 | -2.79 | -3.65 | -0.86 | [8] |
| 15 | 3-hydroxyindole | 50591 | -2.93 | -3.90 | -4.88 | -3.68 | [9] |
| 16 | 3-methoxy-4-phenoxybenzaldehyde | 18433807 | -2.00 | -2.68 | -3.26 | -0.84 | [8] |
| 17 | 4-(chloromethyl)-2-methoxy-1-phenoxybenzene | 23656754 | -1.86 | -2.72 | -3.30 | -0.85 | [8] |
| 18 | 4-aminophenol | 403 | -2.99 | -4.08 | -5.43 | -5.26 | [6] |
| 19 | 4-anisidine | 7732 | -2.60 | -3.56 | -4.57 | -3.56 | [6] |
| 20 | 4-hydroxyindole | 75421 | -2.88 | -3.84 | -4.80 | -3.66 | [9] |
| 21 | 4-sulfonamide-4'-dimethylaminoazobenzene (LB-152) | 75522 | -1.68 | -2.44 | -3.66 | -0.46 | [10] |
| 22 | AC1NYJ6O | 5856764 | -1.48 | -2.02 | -2.60 | -0.23 | [11] |
| 23 | Acid-red-27 | 5464286 | -1.29 | -1.99 | -3.48 | -0.19 | [12] |
| 24 | Anthraquinone | 6780 | -2.67 | -3.48 | -3.97 | -1.47 | [13] |
| 25 | Apigenin | 5280443 | -2.04 | -2.76 | -3.68 | -0.70 | [5] |
| 26 | Apomorphine | 6005 | -2.21 | -2.96 | -3.48 | -0.87 | [14] |
| 27 | Aspirin | 2244 | -2.20 | -3.05 | -4.40 | -1.61 | [15] |
| 28 | Astringin | 5281712 | -1.42 | -1.99 | -2.88 | -0.23 | [16] |
| 29 | Baicalein | 5281605 | -2.09 | -2.83 | -3.77 | -0.74 | [17] |
| 30 | Carvedilol | 2585 | -1.33 | -1.81 | -2.26 | -0.18 | [18] |
| 31 | Catechin | 9064 | -1.84 | -2.54 | -3.56 | -0.60 | [19] |
| 32 | Cotinine | 854019 | -2.34 | -3.17 | -4.12 | -1.73 | [20] |
| 33 | Crocetin | 5281232 | -1.69 | -2.31 | -2.77 | -0.29 | [21] |
| 34 | Curculigoside-B | 132567 | -1.18 | -1.67 | -2.54 | -0.17 | [22] |
| 35 | Curcumin | 969516 | -1.43 | -1.96 | -2.51 | -0.23 | [23,24] |
| 36 | Daunomycin | 30323 | -1.23 | -1.71 | -2.40 | -0.16 | [18] |
| 37 | Dimethyl-Yellow | 6053 | -2.11 | -2.80 | -3.40 | -0.79 | [5] |
| 38 | Doxycycline | 5281011 | -1.19 | -1.66 | -2.41 | -0.23 | [25] |
| 39 | Ectoine | 126041 | -2.29 | -3.26 | -5.43 | -2.79 | [26] |
| 40 | Endomorphin-1 | 5311080 | -1.04 | -1.42 | -1.88 | -0.08 | [27] |
| 41 | Endomorphin-2 | 5311081 | -1.09 | -1.49 | -1.95 | -0.09 | [27] |
| 42 | Entacapone | 5281081 | -1.47 | -2.05 | -3.21 | -0.42 | [28] |
| 43 | Epicatechin-gallate | 107905 | -1.24 | -1.71 | -2.49 | -0.19 | [5,19] |
| 44 | Epsilon-viniferin | 5281728 | -1.42 | -1.89 | -2.30 | -0.20 | [16,29] |
| 45 | Exifone | 40399 | -1.76 | -2.45 | -3.77 | -0.62 | [5] |
| 46 | Fenofibrate | 3339 | -1.50 | -2.16 | -2.70 | -0.31 | [3] |
| 47 | GHK (Gly-L-His-L-Lys) | 73587 | -1.18 | -1.68 | -2.87 | -0.28 | [30] |
| 48 | Glutathione | 124886 | -1.20 | -1.84 | -3.68 | -0.38 | [31] |
| 49 | Gossypetin | 5280647 | -1.75 | -2.42 | -3.71 | -0.51 | [5] |
| 50 | Hydroxyectoine | 12011795 | -2.10 | -3.02 | -5.53 | -2.24 | [26] |
| 51 | Hypericin | 5281051 | -1.59 | -2.11 | -2.67 | -0.24 | [5] |
| 52 | Indole-3-carbinol | 3712 | -2.80 | -3.75 | -4.58 | -2.88 | [9] |
| 53 | Indomethacin | 3715 | -1.63 | -2.33 | -3.06 | -0.41 | [32] |
| 54 | Juglone | 3806 | -2.64 | -3.54 | -4.60 | -2.13 | [3] |
| 55 | Kaempferol | 5280863 | -1.85 | -2.52 | -3.53 | -0.61 | [19] |
| 56 | Kyotorphin | 123804 | -1.45 | -2.04 | -3.27 | -0.31 | [33] |
| 57 | Malvidin | 159287 | -1.56 | -2.16 | -3.05 | -0.41 | [22] |
| 58 | Melanostatin | 92910 | -1.52 | -2.16 | -3.32 | -0.49 | [34] |
| 59 | Melatonin | 896 | -2.08 | -2.84 | -3.71 | -0.86 | [35] |
| 60 | Minocycline | 5281021 | -1.12 | -1.56 | -2.23 | -0.19 | [36] |
| 61 | Morin | 5281670 | -1.77 | -2.44 | -3.57 | -0.55 | [19] |
| 62 | Myricetin | 5281672 | -1.67 | -2.30 | -3.53 | -0.48 | [19,37] |
| 63 | Neocuproine | 65237 | -2.57 | -3.35 | -3.83 | -1.35 | [3] |
| 64 | Nordihydroguaiaretic acid | 71398 | -1.77 | -2.43 | -2.97 | -0.45 | [19] |
| 65 | Oenin | 443652 | -1.08 | -1.53 | -2.32 | -0.15 | [22] |
| 66 | O-Ethylvanillin | 68117 | -2.25 | -3.12 | -4.16 | -1.89 | [6] |
| 67 | Oxyquinoline | 1923 | -2.92 | -3.85 | -4.71 | -3.03 | [38] |
| 68 | Perphenazine | 4748 | -1.27 | -1.90 | -2.44 | -0.26 | [5] |
| 69 | Phenolphthalein | 4764 | -1.78 | -2.36 | -2.83 | -0.49 | [39] |
| 70 | Phenolsulfonphthalein | 4766 | -1.60 | -2.26 | -2.98 | -0.44 | [39] |
| 71 | Phthalocyanine | 5282330 | -1.77 | -2.30 | -2.88 | -0.20 | [5] |
| 72 | Piceatannol | 667639 | -2.10 | -2.86 | -3.67 | -0.76 | [16] |
| 73 | Piceid | 5281718 | -1.49 | -2.07 | -2.90 | -0.26 | [16,29] |
| 74 | Protocatechuic-acid | 72 | -2.61 | -3.65 | -5.74 | -2.64 | [6] |
| 75 | Pseudohypericin | 5281751 | -1.48 | -1.98 | -2.57 | -0.22 | [5] |
| 76 | Quercetin | 5280343 | -1.82 | -2.50 | -3.67 | -0.56 | [19,28] |
| 77 | Quinacrine | 237 | -1.23 | -1.75 | -2.13 | -0.23 | [5] |
| 78 | Resveratrol | 445154 | -2.22 | -2.98 | -3.61 | -0.87 | [16,29] |
| 79 | Rhodamine-B | 45271881 | -1.27 | -1.70 | -1.93 | -0.19 | [3] |
| 80 | Rolitetracycline | 54679406 | -1.08 | -1.50 | -2.11 | -0.14 | [18] |
| 81 | Rolitetracycline | 54682938 | -1.08 | -1.50 | -2.11 | -0.14 | [18] |
| 82 | Rosmarinic acid | 5281792 | -1.42 | -1.96 | -2.83 | -0.26 | [40] |
| 83 | ScirpusinA | 5458896 | -1.35 | -1.81 | -2.26 | -0.18 | [16,29] |
| 84 | Scyllo-inositol | 892 | -1.92 | -2.88 | -5.77 | -1.99 | [41] |
| 85 | Serotonin | 5202 | -2.39 | -3.25 | -4.22 | -1.77 | [42] |
| 86 | Silybin | 31553 | -1.32 | -1.82 | -2.55 | -0.17 | [43] |
| 87 | Tetramethoxycurcumin | 9952605 | -1.27 | -1.74 | -2.19 | -0.18 | [11] |
| 88 | Tocopherylquinone | 24205 | -1.11 | -1.56 | -1.72 | -0.13 | [44] |
| 89 | Tolcapone | 4659569 | -2.00 | -2.73 | -3.90 | -0.67 | [28] |
| 90 | Tramiprosate | 1646 | -1.88 | -3.28 | -8.73 | -3.69 | [45] |

[1] Campagna F, Catto M, Purgatorio R, Altomare CD, Carotti A, et al. (2011) Eur J Med Chem 46: 275-284. [2] Skovronsky DM, Zhang B, Kung MP, Kung HF, Trojanowski JQ, et al. (2000) Proc Natl Acad Sci U S A 97: 7609-7614. [3] Necula M, Kayed R, Milton S, Glabe CG (2007) J Biol Chem 282: 10311-10324. [4] Simons LJ, Caprathe BW, Callahan M, Graham JM, Kimura T, et al. (2009) Bioorg Med Chem Lett 19: 654-657. [5] Taniguchi S., Suzuki N., Masuda M., Hisanaga S., Iwatsubo T., et al. (2005) J Biol Chem 280: 7614-7623. [6] De Felice FG, Vieira MN, Saraiva LM, Figueroa-Villar JD, Garcia-Abreu J, et al. (2004) FASEB J 18: 1366-1372. [7] Soto-Ortega DD, Murphy BP, Gonzalez-Velasquez FJ, Wilson KA, Xie F, et al. (2011) Bioorg Med Chem 19: 2596-2602. [8] Gupta S, Babu P, Surolia A (2010) Biomaterials 31: 6809-6822. [9] Cohen T, Frydman-Marom A, Rechter M, Gazit E (2006) Biochemistry 45: 4727-4735. [10] Lin SJ, Shiao YJ, Chi CW, Yang LM (2004) Bioorg Med Chem Lett 14: 1173-1176. [11] Orlando RA, Gonzales AM, Royer RE, Deck LM, Vander Jagt DL (2012) PLoS One 7: e31869. [12] Pollack SJ, Sadler II, Hawtin SR, Tailor VJ, Shearman MS (1995) Neurosci Lett 197: 211-214. [13] Convertino M, Pellarin R, Catto M, Carotti A, Caflisch A (2009) Protein Sci 18: 792-800. [14] Lashuel HA, Hartley DM, Balakhaneh D, Aggarwal A, Teichberg S, et al. (2002) J Biol Chem 277: 42881-42890. [15] Hirohata M, Ono K, Naiki H, Yamada M (2005) Neuropharmacology 49: 1088-1099. [16] Rivière C, Richard T, Quentin L, Krisa S, Mérillon JM, et al. (2007) Bioorg Med Chem 15: 1160-1167. [17] Lu JH, Ardah MT, Durairajan SS, Liu LF, Xie LX, et al. (2011) Chembiochem 12: 615-624. [18] Howlett DR, George AR, Owen DE, Ward RV, Markwell RE (1999) Biochem J 343: 419-423. [19] Ono K, Yoshiike Y, Takashima A, Hasegawa K, Naiki H, et al. (2003) J Neurochem 87: 172-181. [20] Echeverria V, Zeitlin R, Burgess S, Patel S, Barman A, et al. (2011) J Alzheimers Dis 24: 817-835. [21] Ahn JH, Hu Y, Hernandez M, Kim JR (2011) Biochem Biophys Res Commun 414: 79-83. [22] Rivière C, Richard T, Vitrac X, Mérillon JM, Valls J, et al. (2008) Bioorg Med Chem Lett 18: 828-831. [23] Doggui S, Sahni JK, Arseneault M, Dao L, Ramassamy C (2012) J Alzheimers Dis 30: 377-392. [24] Yang F, Lim GP, Begum AN, Ubeda OJ, Simmons MR, et al. (2005) J Biol Chem 280: 5892-5901. [25] Forloni G, Colombo L, Girola L, Tagliavini F, Salmona M (2001) FEBS Lett 487: 404-407. [26] Kanapathipillai M, Lentzen G, Sierks M, Park CB (2005) FEBS Lett 579: 4775-4780. [27] Frydman-Marom A, Convertino M, Pellarin R, Lampel A, Shaltiel-Karyo R, et al. (2011) ACS Chem Biol 6: 1265-1276. [28] Di Giovanni S, Eleuteri S, Paleologou KE, Yin G, Zweckstetter M, et al. (2010) J Biol Chem 285:14941-14954. [29] Rivière C, Papastamoulis Y, Fortin PY, Delchier N, Andriamanarivo S, et al. (2010) Bioorg Med Chem Lett 20: 3441-3443. [30] Guilloreau L, Combalbert S, Sournia-Saquet A, Mazarguil H, Faller P (2007) Chembiochem 8: 1317-1325. [31] Woltjer RL, Nghiem W, Maezawa I, Milatovic D, Vaisar T, et al. J Neurochem 93: 1047-1056. [32] Netland EE, Newton JL, Majocha RE, Tate BA (1998) Neurobiol Aging 19:201-204. [33] Zakutskiĭ AN, Chalisova NI, Subbotina TF (2008) Bioorg Khim 34: 149-159. [34] Gasperini RJ, Small DH (2012) Clin Exp Pharmacol Physiol 39: 680-683. [35] He H, Dong W, Huang F (2010) Curr Neuropharmacol 8: 211-217. [36] Familian A, Boshuizen RS, Eikelenboom P, Veerhuis R (2006) Glia 53: 233-240. [37] Hirohata M, Hasegawa K, Tsutsumi-Yasuhara S, Ohhashi Y, Ookoshi T, et al. (2007) Biochemistry 46: 1888-1899. [38] Adlard PA, Cherny RA, Finkelstein DI, Gautier E, Robb E, et al. (2008) Neuron 59:4 3-55. [39] Levy M, Porat Y, Bacharach E, Shalev DE, Gazit E (2008) Biochemistry 47: 5896-5904. [40] Ono K, Yamada M (2006) J Neurochem 97: 105-115. [41] McLaurin J, Kierstead ME, Brown ME, Hawkes CA, Lambermon MH, et al. (2006) Nat Med 12: 801-808. [42] Cirrito JR, Disabato BM, Restivo JL, Verges DK, Goebel WD, et al. (2011) Proc Natl Acad Sci U S A 108: 14968-14973. [43] Yin F, Liu J, Ji X, Wang Y, Zidichouski J, et al. (2011) Neurochem Int 58: 399-403. [44] Yang SG, Wang WY, Ling TJ, Feng Y, Du XT, et at. (2010) Neurochem Int 57: 914-922. [45] Wright TM (2006) Drugs Today (Barc) 42: 291-298.
